# Acute Left Atrial Response to Different Eccentric Resistance Exercise Loads in Patients with Heart Failure with Middle Range Ejection Fraction: A Pilot Study

**DOI:** 10.3390/jpm12050689

**Published:** 2022-04-26

**Authors:** Giuseppe Caminiti, Marco Alfonso Perrone, Ferdinando Iellamo, Valentino D’Antoni, Matteo Catena, Alessio Franchini, Maurizio Volterrani

**Affiliations:** 1 Cardiology Rehabilitation Unit, IRCCS San Raffaele Pisana, 00163 Rome, Italy; giuseppe.caminiti@sanraffaele.it (G.C.); iellamo@uniroma2.it (F.I.); valentino.dantoni@sanraffaele.it (V.D.); matteo.catena.94@gmail.com (M.C.); franchini.fkt@gmail.com (A.F.); maurizio.volterrani@sanraffaele.it (M.V.); 2 Department of Clinical Sciences and Translational Medicine, University of Rome Tor Vergata, 00133 Rome, Italy

**Keywords:** resistance exercise, heart failure, atrial function, eccentric exercise, ventricular strain

## Abstract

In this study, we aimed to assess acute changes occurring on atrial function following single bouts of eccentric resistance exercise (ECC-RE) performed at two different loads. Twenty-five patients with chronic heart failure with middle range ejection fraction (HFmrEF) participated in three experimental sessions in a randomized order and on separate days: two sessions of ECC RE at 20% (ECC-20) of one-repetition maximum (1-RM) and 50% (ECC-50) 1-RM, and one session of control, without exercise. Each session lasted three minutes. Before and immediately after the sessions, patients underwent echocardiography and blood pressure and heart rate measurement. Peak atrial longitudinal strain (PALS) and peak atrial contractile strain (PACS) significantly increased after both ECC-20 (+16.3%) and ECC-50 (+18.1%) compared to control (between sessions *p* = 0.022). Peak atrial contractile strain (PACS) significantly increased after ECC-50 (+28.4%) compared to ECC-20 (+17.0%) and control (between sessions *p* = 0.034). The ratio of transmitral and annular velocities (E/E’) increased significantly after ECC-20 (+10.4%) and ECC-50 (+19.0%) compared to control (between groups *p* = 0.003). EF, left ventricular longitudinal strain, and stroke volume did not change after ECC-RE sessions compared to control. Cardiac output increased significantly after ECC-20 and ECC-50 compared to control, (between groups *p* = 0.025). In conclusion, both ECC-RE sessions were well tolerated, and LA functional reserve was properly mobilized in response to ECC-RE in patients with HFmrEF. Cardiac output increased at the cost of an increased LV filling pressure, but no detrimental changes of LV function occurred.

## 1. Introduction

Exercise intolerance is a cardinal symptom of heart failure (HF), and it represents an independent predictor of poor prognosis in patients suffering from this disease [1]. Physical exercise is a non-pharmacological treatment for HF, and it has a well-established impact on symptoms, quality of life and disease progression [2]. While aerobic training is the most recommended exercise modality for HF patients, the addition of isokinetic resistance exercise (RE) in their training programs has progressively gained a growing interest in the last decades and has been included into cardiac rehabilitation [3]. Isokinetic RE induces peripheral muscles hypertrophy, increases muscle strength [4], prevents decline in skeletal muscle mass [5] and exerts a positive impact on daily life activities and quality or life, especially in older individuals [6,7]. Direct cardiac effects have also been described for isokinetic RE; in patients with previous myocardial infarction, it attenuated left ventricular (LV) dilation and dysfunction [8]. Among different subtypes of isokinetic RE, eccentric, as opposed to concentric, resistance exercise (ECC-RE) appears particularly suitable for elderly patients with HF and has been shown to result in greater increase in muscle strength, while at the same time, it reduces oxygen requirement and cardiovascular stress in comparison to concentric exercise [9]. However, uncertainty still exists about which is the best ECC prescription for HF patients in terms of exercise load, number of repetitions and safety of exercise protocols. In order to clarify which ECC protocol is better tolerated by HF patients and to develop more effective, and safe, exercise regimens, it appears useful to evaluate cardiac adaptations to this type of exercise. LV changes occurring during resistance training have been widely studied both in healthy subjects and in patients with HF [10,11,12,13,14,15,16]. However, the cardiac response to RE in patients with heart failure with middle range EF (HFmrEF) has been less investigated. Moreover, there are not data available regarding the left atrial (LA) functional response to RE in patients with HF and, in particular, in those with HFmrEF. The left atrium is a hemodynamic mediator between LV and pulmonary circulation. It contributes to LV filling and protects the pulmonary capillary bed from pressure and volume overloads [17]. Therefore, investigating LA dynamics during exercise plays a pivotal role in the understanding of mechanisms of exercise intolerance in HF patients and can help to find more suitable exercise protocols for training these patients. To date, acute and long-term changes in LA function elicited by exercise have been evaluated mostly in healthy subjects and athletes, with inconsistent results [18,19]. A recent study has shown that in patients with HF, the acute LA functional response during an exercise ramp test is blunted compared to control [20]. However, several aspects remain to be clarified. It is unknown whether the atrial response described by Sugimoto et al. [20] can be generalized to other exercise modalities; moreover, it is possible that the atrial response to a given exercise varies in relation to the type of HF. In this study, we aimed to characterize the LA functional response to resistance exercise in patients with HFmrEF. In particular, we investigated acute changes in LA function in response to single sessions of ECC-RE performed at different loads. Changes in diastolic and systolic LV function were also investigated.

## 2. Methods

### 2.1. Population

The study included 25 patients referred to a cardiac rehabilitation program at the cardiac rehabilitation center in San Raffaele IRCCS, Rome, between June 2020 and March 2021. Participants of this study were sent to our cardiac rehabilitation facility by their own family doctor or private cardiologist. Inclusion criteria were as follows: diagnosis of HFmrEF (EF between 41% and 49%) with underlying coronary artery disease, stable clinical conditions and stable pharmacological therapy (not being modified in the last three months), sedentary lifestyle (patients not being enrolled in exercise training programs in the previous six months) and sinus rhythm. Exclusion criteria were: BP levels exceeding 160/100 mmHg, heart valve diseases, hypertrophic cardiomyopathy, signs and/or symptoms of myocardial ischemia during a preliminary ergometric test, uncontrolled arrhythmia, neurological and/or orthopedic conditions contraindicating or limiting exercises, significant chronic obstructive pulmonary disease (i.e., FEV1 < 50%), and symptomatic peripheral arterial disease. For the purpose of the study, the diagnostic criteria for coronary artery disease were: previous myocardial infarction, a history of percutaneous coronary interventions and/or coronary artery bypass graft.

### 2.2. Study Design

**Figure 1:**
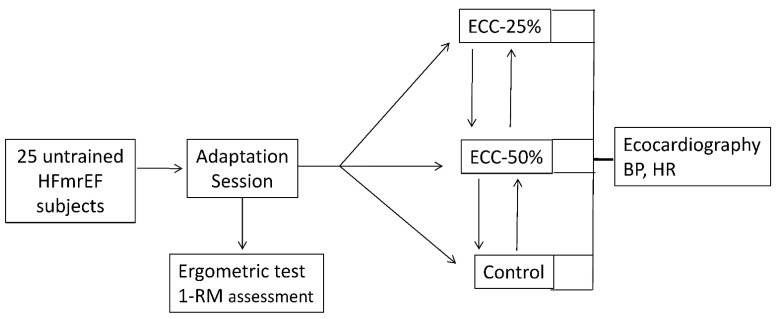
Study flowchart.

The study flowchart is reported in Figure 1. The study had a cross-over design. It was preliminarily approved by the ethical committee of San Raffaele IRCCS, Rome (prot. n. 24/20). All patients gave informed consent to participate in the study, which was conformed to the principles outlined in the Declaration of Helsinki. In this study, we chose leg extension/flexion as the isokinetic RE modality, since in this type of exercise, the chest is stable and the echocardiographic windows enable the acquisition of high quality data. In a preliminary visit, all patients underwent a baseline evaluation, including clinical and pharmacological history and the assessment of body mass index (BMI). Subsequently, they performed a symptoms-limited ergometric test in order to rule out exercise-induced ischemia and ventricular arrhythmias during exercise. The ergometric test was performed on a treadmill (Mortara Instr, Bologna, Italy), and a standard modified Bruce protocol was adopted. In the same day, patients performed an adaptative session during which they were familiarized with the gym equipment and the established exercise modality. At the end of this adaptative session, patients underwent the assessment of 1-repetition maximum (1-RM) for leg flexion. This test was performed from the sitting position on an isokinetic dynamometer (Technogym Wellness System, Cesena, Italy). During the test, patients were first instructed to complete a warm-up set comprising eight repetitions at 50% and six repetitions at 70% of their perceived 1-RM. The load was then progressively increased until reaching the workload that could be lifted between three and five times (3–5 RM), with 2–3 min rest between efforts [21]. Then, 1-RM was calculated using a prediction equation [22]. Afterward, every patient performed three experimental sessions. The order of these sessions was randomly assigned through computer software.

The experimental sessions were performed in the morning, between 9:30 and 11:00, on different days. Before testing, patients were instructed to abstain from exercise in the previous 24 h. Moreover they were asked to not smoke, and not drink coffee and alcohol. Patients were allowed to have a light breakfast at least 2 h before the start of the sessions and to regularly take their morning drugs. Before starting the ECC-RE, all participants performed a 5 min warm-up on a stationary bike. Then, they were required to perform leg extension/flexion movements from the sitting position upon the isokinetic; the hip and thigh were carefully fastened to the seat, with both legs attached to the dynamometer lever arm about 2 cm above the medial malleolus, with both arms along the trunk, and with hands holding the bars on the side of the chair. Axis speed and range of movement were fixed at 10° s^−1^ and 90°, respectively. Patients performed, in a random order, a session of ECC-RE at 20% of their ECC 1-RM (ECC-20), a session of ECC-RE at 50% of their ECC 1-RM (ECC-50), or a control session. Every session lasted three minutes, and by keeping a constant rate during the exercises, patients were required to perform 45 repetitions in each session. After the first twenty-five repetitions, patients rested for 30 s and then completed the remaining 20 repetitions. During the control session, patients rested for three minutes in a seated position on the chair of the dynamometer. In order to obtain a pure eccentric exercise, no loads were applied during the concentric component of the movement. The exercise intensities and duration were based on previous studies that used eccentric exercise [23] and enrolled patients with cardiovascular diseases [14]. Echocardiography was started within one minute from the end of each exercise session, while BP (and heart rate) was measured in the dominant arm by using an oscillometric automatic device (OMRON, Healthcare Inc., Milan, Italy). Rate of perceived exertion (RPE) was assessed by using the Borg 6–20 scale [24]. Each exercise lasted three minutes. During the experimental sessions, patients were monitored through a three-lead ECG connected to the echocardiogram. 

### 2.3. Assessments

*Echocardiography:* All echocardiographic examinations were made using Acuson SC 2000 Prime ultrasound system (Siemens) with a 4.0 MHz transducer by one experienced sonographer who was blinded to the type of experimental sessions performed by individual patients. Echocardiography was performed before starting the exercise (T0) and immediately after the end of the exercise (T1). In the case of control sessions, echocardiography was performed before and after three minutes of rest. Left ventricular end-diastolic volume (LVEDV) and end-systolic volume (LVESV) were calculated from the apical two and four chamber windows using modified Simpson’s method, which was used to calculate stroke volume (SV) as EDV−ESV, cardiac output (CO) as HR × SV, and ejection fraction (EF) as EF = (EDV−ESV)/EDV). LA volume was measured from standard apical 4-chamber views at end systole directly before mitral valve opening. The biplane method of disks was used to calculate LA volume. LAVi was calculated by dividing LA volume by body surface area of the subjects. The reported E/A ratio represents the ratio of peak left ventricle filling velocity in early diastole (E wave) to that in late diastole during atrial contraction (A wave). LV E/E^1^ ratio was calculated as the ratio between E wave velocity and mean lateral and septal LV E^1^ wave velocities. Color tissue Doppler tracings were obtained with the range gate placed at the lateral mitral annular segments in the 4-chamber view. Peak systolic LV longitudinal strain and strain rates were assessed using standard 2D apical four chambers and two chambers view using speckle-tracking analysis. Global longitudinal strain (GLS) was determined by averaging all values of the segments of the two views. LA strain was measured from the 4-chamber and 2-chamber views. The software program generated the longitudinal strain curves for each segment and a mean curve of all segments. The 3-chamber apical view was not included for the GLS calculation because of the poor quality of images obtained in the seated position. Peak atrial longitudinal strain (PALS) was measured at the end of the reservoir phase (positive peak during LV systole), and peak atrial contraction strain (PACS) was measured directly before the start of the active contractile phase (positive peak during early diastole). LV stiffness was calculated as E/E^1^/LVEDV [25]. LA stiffness was calculated as E/E^1^/PALS [26]. Rate of pressure product was calculated as index of myocardial oxygen consumption according to the formula: RPP = (HR × SBP)/100

### 2.4. Statistical Analysis

Since we did not find in the literature previous direct comparisons between two different eccentric loads on LA function, we were not able to calculate the study sample size, and therefore, this research was conceived as a pilot study. Data are expressed as mean ± SD. The assumption of normality was checked using Shapiro–Wilks hypothesis test. Pre- and post-exercise data of normally distributed variables were assessed using repeated measures two-way ANOVA, with Bonferroni corrections for post hoc testing. Not normally distributed variables were assessed using Kruskal–Wallis test and Bonferroni corrections for post hoc testing. The level of significance was set at *p* < 0.05. Data were analyzed using SPSS software (version 20.0 IBM Corp, Amonk, New York, NY, USA).

## 3. Results

Anthropometric and clinical features of patients are summarized in Table 1. Eighteen out of twenty-five (72%) were overweight, and three (12%) were obese. Sixteen out of twenty-five (64%) had a previous myocardial infarction. Men and women had similar average age and BMI (men at 70.8 ± 9.3 and 27.1 ± 8.0, respectively; women at 71.9 ± 5.2 and 27.7 ± 6.8, respectively). Women had a lower rate of betablockers than males (40% and 80%, respectively). All patients completed the task of performing 45 repetitions during three minutes of exercise at 20% and 50% ECC. The Borg’s scale score at the end of ECC sessions was 9.4 ± 2.0, corresponding to very light after ECC-20 and 13.2 ± 2.8 corresponding to somewhat hard after ECC-50. All patients completed the study, and there were no drop-outs.

**Figure 2:**
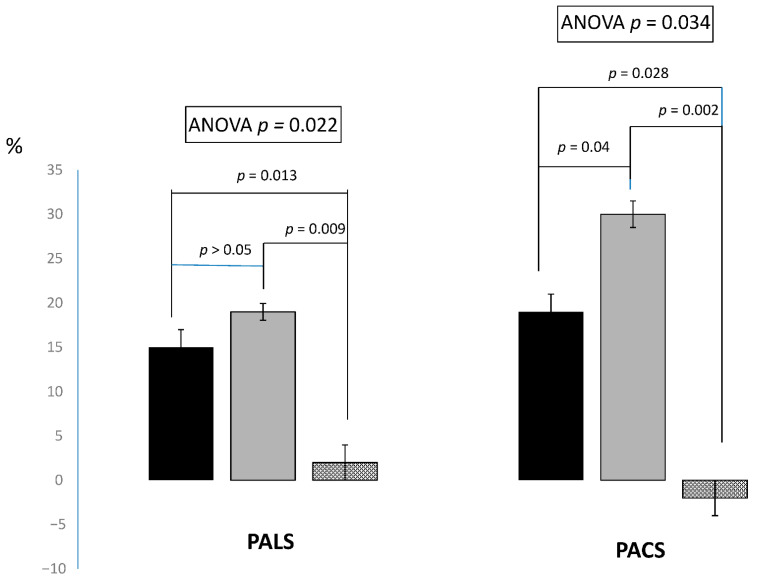
Percentage changes of PALS and PACS after ECC-20 (black-bars), ECC-50 (gray-bars) and control session (dotted bars). Results of ANOVA and post hoc testing.

Changes in hemodynamic and echocardiographic parameters are summarized in Table 2. Systolic BP and MAP increased significantly after both exercise sessions and remained unchanged after control (between sessions *p* = 0.013 for systolic BP and *p* = 0.039 for MAP). There were not significant differences in changes of systolic BP between ECC-20 and ECC-50. Diastolic BP did not change after exercise sessions compared to control. HR increased after both exercise sessions and was unchanged in control session (between sessions *p* = 0.047). PALS increased in a similar manner after ECC-20 and ECC-50 and was unchanged after control session (between sessions *p* = 0.022). PACS increased significantly after both exercise sessions compared to control (between sessions *p* = 0.034). The increase in PACS after ECC-50 was also significantly higher than after ECC-20 (Figure 2). A’ wave increased significantly after both ECC-20 and ECC-50 compared to control session (between groups *p* = 0.038). LAVI and LA stiffness were unchanged after both exercise sessions and control (Table 2). SV did not change after exercise sessions compared to control. CO increased after both exercise sessions (between group *p* = 0.025) and remained unchanged after control. E/E’ increased after both ECC-20 (+0.9 ± 0.2) and ECC-50 (+1.5 ± 0.3) and remained unchanged after control session (between groups *p* = 0.003).

Overall, both exercise sessions were well tolerated, and all patients completed their tasks. None of them reported any discomfort during RE, and no adverse events occurred during the study.

## 4. Discussion

In this study, we investigated LA functional response to a single bout of two different intensities of ECC-RE in patients with HFmrEF. We found that PALS and PACS significantly increased after both ECC-20 and ECC-50, with PACS having the greatest increase with the highest volume. To the best of our knowledge, this is the first study to assess acute LA functional response to different eccentric volumes in HFmrEF patients. LA function has relevant importance in the pathophysiology of HF and plays a key role in modulating LV filling in these patients, particularly during exercise [27]. To date, acute exercise-induced changes in LA function have been investigated mostly in animal models [28] and healthy subjects [29], and increases in both LA reservoir and booster functions during exercise have been demonstrated. However, the exercise-mediated LA response seems to be impaired in pathological conditions; available data show that it is depleted in subjects with advanced heart disease and heart remodeling. In patients with HF with reduced EF performing a ramp incremental test, LA reservoir and booster functions were impaired both at peak exercise and during recovery when compared to healthy controls [20]. In the study of Tan et al. [30], the possibility to mobilize atrial functional contractile reserve in response to exercise was preserved in hypertensive subjects, while it was lost in patients with HF with preserved EF. In this study, specific characteristics of the exercise modality applied and the degree of LA elastic compliance might explain the type of LA functional response that we observed. Contrary to endurance exercise, resistance exercise is associated with modest or no increase in venous return [31], and this might have avoided overstretching of the LA beyond its elastic properties during our experiments; at the same time, this limited increase in venous return did not cause a clinically significant rise in LV filling pressure. Although we observed an increase in E/E’ ratio after exercise sessions versus control, E/E’ resting values were at the upper limits of the normal range, and the increases observed after the two exercise sessions were well below the threshold usually considered as indicative of diastolic dysfunction [32,33]. Therefore, we can assume that the mild increase in LV filling pressure registered had no detrimental consequences on LA reservoir function. Although PALS values at rest were lower than those reported for healthy subjects, indicating a partially compromised LA reservoir function [34], the increase in PALS during ECC-RE suggests that the elastic properties of LA were, at least in part, preserved. The ECC-RE-mediated expansion of the reservoir phase might have, in turn, promoted an increase in the LA booster function according to a Frank–Starling mechanism as described by Ashraf et al. [35]. When compared with previous research, the increase in PALS and PACS after ECC-RE observed in the present study suggests that acute LA response to exercise could vary in relation to different exercise modalities and loads. This result is in agreement with another recent study, in which PALS and PACS increased, albeit not significantly, after a session of concurrent, aerobic plus resistance, exercise, while they were significantly decreased after a session of high-intensity interval exercise in subjects with hypertension and ischemic heart disease [36]. We observed that PALS increased in a similar manner after ECC-20 and ECC-50, while PACS increased to a greater extent after ECC-50 (+28.4%) than after ECC-20 (+18.1%). We speculate that the functional reserve put in place by the reservoir phase after ECC-20 was already at its maximum limit and could not be further expanded. Conversely, the LA contractile reserve (booster phase) was proportional to the external load, and its relative contribution in maintaining LV filling became preponderant during ECC-50.

In this study, we observed a statistically significant increase in CO after both ECC-20 and ECC-50 versus control. This result suggests that patients with HFmrEF were able to increase CO in response to mild-to-moderate external ECC-RE loads at the price of a mild increase in LV filling pressure. Since SV did not change significantly after both ECC-20 and ECC-50 compared to control, the increase in CO observed in our study was mainly due to the increase in HR. The hemodynamic profile that we found during ECC-RE differed from the findings of Stohr et al. [10] performed in healthy subjects, in which there were no significant changes in diastolic parameters during RE. On the contrary, our results are in line with those of previous studies performed in patients with CHF [12,37,38], which showed similar hemodynamic responses to different exercise modalities. However, our results on SV differed from those obtained by Cheetham et al. [12], who reported a decrease in SV after RE in patients with more advanced HF. Differences in CHF stage and exercise intensities could explain these apparently inconsistent results. The average EF of our patients 46.7 ± 4.3, whereas it was much lower in the study by Cheetham et al. [12]; hence, it is possible that the greater severity of the disease in the study by Cheetham et al. [12] could have resulted in a more severely blunted LA functional reserve in comparison to our study. In addition to SV, we found that other indices of LV function as LVEF and GLS were unchanged after both ECC-20 and ECC-50. These findings indicate that the observed rise in LV filling pressure after ECC-RE was not sufficient to elicit detrimental changes on LV function. This result is in line with a similar study conducted in healthy subjects, in which indices of LV function, including LV longitudinal strain, were unchanged during ECC-RE performed at different loads [11]. However, different data have been observed in healthy subjects. Stefani et al. [39] reported improvements in apical longitudinal strain in athletes during a handgrip exercise for 3 min at 30% of their maximal contraction. Conversely, other authors have observed reductions in LV strain during handgrip [40] or leg press exercises [10]. The type of population—in particular, the presence or not of cardiac diseases, exercise protocols adopted and pharmacological treatments—might have affected the results of these studies. The lack of agreement between studies underlines the need for further research evaluating LV strain during RE in HF patients.

We observed that HR and SBP increased after ECC-20 and ECC-50 compared to control. The extent of the increase in SBP was similar between the two RE volumes. In our study, patients performed two sessions of RE at different loads, while time of exercise (3 min) was constant and thus was the number of repetitions/sessions. Therefore, our results suggest that SBP response was not related to the magnitude of the external load. Our data are in agreement with previous research performed in healthy subjects and in patients with ischemic heart disease in which the main determinant of the SBP response was the number of repetitions [13,41].

*Limitations.* The most important limitation of this study is the small sample size; further larger studies are needed in order to clarify atrial changes in response to different RE loads. We evaluated LA response to eccentric loads in HFmrEF patients; considering the study design and the lack of data in this particular population, our study does not clarify whether the observed response is exclusive of ECC-RE or if it can also be obtained with other exercise modalities such as concentric resistance exercise. Another limitation is the lack of a control group without cardiovascular diseases, and thus, a “normal” response to our protocol cannot be determined. Our results do not establish whether the type of LA response observed is specific to eccentric RE or if it can be extended to concentric RE; new studies comparing LA response to concentric and eccentric RE are needed. All hemodynamic parameters were measured or calculated by echocardiography, and therefore, the study lacks comparison with invasive hemodynamic assessment through cardiac catheterization. This study enrolled only patients with HFmrEF and underlying ischemic heart disease; therefore, our results cannot be extended to patients with different etiology and/or disease severity. 

## 5. Conclusions

In patients with HFmrEF, the hemodynamic response to single ECC-RE session was characterized by a proper increase in LA function and CO without detrimental changes in LV function, albeit at the cost of a modest rise in LV filling pressure. New studies evaluating LA response to different exercise intensities and modalities are needed in order identify the most suitable exercise for the rehabilitation programs of patients with HFmrEF.

## Figures and Tables

**Table 1:** Anthropometric and clinical characteristics of patients.

Age, years	71.4 ± 7.5
BMI, kg/m^2^	27.5 ± 6.3
Waist circumference, cm	104.2 ± 31.6
Male/female	20/5
Previous PCI/CABG	16/14
NYHA class (II-III)	19/6
EF (%)	46.7± 6.2
NT-pro BNP	465.0 ± 59.3
*Comorbidities*	
Carotid artery disease, *n* (%)	13 (52)
Hypertension, *n* (%)	25/(100)
Diabetes, *n* (%)	6 (24)
Hypercholesterolemia, *n* (%)	21 (84)
Previous Smoke habit, *n* (%)	18 (60)
*Treatment*	
Anti-platelets agents, *n* (%)	25 (100)
ACE-Is/ARBs, *n* (%)	22 (88)
Betablockers, *n* (%)	18 (60)
Diuretics, *n* (%)	8 (32)
Statins, *n* (%)	25 (100)

BMI, body mass index; EF, ejection fraction; ACE-Is, angiotensin-converting enzyme inhibitors; ARBs, angiotensin receptor blockers.

**Table 2:** Hemodynamic and echocardiography parameters before (T0) and after (T1) exercise sessions.

	ECC-20%		ECC-50%		Control	
	T0	T1	T0	T1	T0	T1
**HR, bpm**	61.2 ± 22.4	68.6 ± 23.2 *	64.5 ± 27.5	71.2 ± 31.8 *	64.1 ± 26.6	66.2 ± 25.7
**SBP, mmHg 1**	113.6 ± 28.2	132.7 ± 25.6 *	107.8 ± 23.5	133.7 ± 19.4 *	109.2 ± 39.6	107.4 ± 28.4
**DBP, mmHg**	74.1 ± 11.5	78.4 ± 10.4	73.6 ± 17.1	75.5 ± 10.8	73,9 ± 13.3	74.0 ± 15.8
**MAP, mmHg**	87.8 ± 11.6	94.8 ± 9.6 *	87.2 ± 13.7	96.3 ± 11.1 *	87.2 ± 13.7	87.2 ± 13.7
**DP, mmHg**	69.5 ± 13.3	91.0 ± 22.1	68.4 ± 17.7	95.2 ± 19.4	68.3 ± 20.5	68.4 ± 16.9
**LA PALS, %**	28.2 ± 8.2	32.7 ± 11.6 *	28.2 ± 10.1	33.5 ± 8.4 *	28.2 ± 10.1	28.6 ± 7.3
**LA PACS, %**	14.0 ± 3.6	16.7 ± 3.8 *	14.2 ± 3.7	18.5 ± 4.6 * ^†^	14.5 ± 2.9	14.3 ± 3.4
**LAVI**	33.4 ± 5.1	32.9 ± 3.9	33.2 ± 4.7	33.5 ± 5.0	33.2 ± 4.3	33.6 ± 6.5
**EDV, mL**	125.6 ±34.5	123.4 ± 40.4	123.7 ± 36.2	126.2 ± 26.9	125.9 ± 30.4	126.0 ± 28.7
**ESV, mL**	67.2 ± 26.3	62.9 ± 24.9	62.3 ± 18.4	65.3 ± 22.1	66.4 ± 19.5	67.1 ± 20.8
**E, cm/s**	57.7 ± 17.88	71.6 ± 21.3	55.6 ± 18.7	71.2 ± 15.6	57.1 ± 16.0	58.3 ± 17.1
**A, cm/s**	59.4 ± 22.6	68.5 ± 16.8	58.3 ± 19.5	73.3 ± 16.2	58.4 ± 19.2	61.2 ± 21.0
**E’**	6.7 ± 1.5	7.5 ± 2.2 *	7.0 ± 1.9	7.5 ± 1.4 *	6.6 ± 2.0	6.8 ± 1.8
**A’**	5.8 ± 1.4	7.1 ± 3.0 *	5.4 ± 1.4	7.8 ± 3.0 *	56 ± 1.9	5.8 ± 2.2
**E/E’**	8.6 ± 1.7	9.5 ± 1.9 *	7.9 ± 2.5	9.4 ± 1.4 *	8.3 ± 2.2	8.1 ± 1.9
**LA stiffness**	0.30 ± 0.2	0.29 ± 0.4	0.28 ± 0.2	0.28 ± 0.3	0.29 ± 0.4	0.28 ± 0.3
**LV stiffness**	68.9 ± 8.3	76.3 ± 10.5 *	63.8 ± 11.8	74.4 ± 12.0 *	65.9 ± 15.2	64.2 ± 9.7
**EF, %**	46.2 ± 3.6	46.8 ± 2.8	45.9 ± 4.5.	46.1 ± 3.3	46.3 ± 5.1	46.1 ± 3.9
**LV GLS, %**	−13.8 ± 1.7	−16.3 ± 2.2	−13.1 ± 1.8	−14.6 ± 2.4	−13.4 ± 2.4	−13.5 ± 1.9
**SV, mL**	58.4 ± 17.3	56.1 ± 11.0	61.4 ± 19.2	62.8 ± 16.8	61.4 ± 13.2	60.9 ± 18.7
**CO, l/min**	3.5 ± 1.5	4.1 ± 1.3 *	3.7 ± 1.2	4.2 ± 1.8 *	3.6 ± 1.7	3.7 ± 1.5

HR, heart rate; SBP, systolic blood pressure; DBP, diastolic blood pressure; MAP, mean arterial pressure; EDV, end-diastolic volume; ESV, end-systolic volume; PALS, peak atrial longitudinal strain; PACS, peak atrial contraction strain; GLS, global longitudinal strain; SV, stroke volume; CO, cardiac output. * *p* < 0.05 vs. control. ^†^ *p* < 0.05 vs. intervention.

## Data Availability

The data presented in this study are available on request from the corresponding author.

## References

[1] Del Buono M.G., Arena R., Borlaug B.A., Carbone S., Canada J.M., Kirkman D.L., Garten R., Rodriguez-Miguelez P., Guazzi M., Lavie C.J. (2019). Exercise Intolerance in Patients with Heart Failure: JACC State-of-the-Art Review. J. Am. Coll Cardiol..

[2] Cattadori G., Segurini C., Picozzi A., Padeletti L., Anzà C. (2018). Exercise and heart failure: An update. ESC Heart Fail..

[3] Franklin B.A., Bonzheim K., Gordon S., Timmis G.C. (1991). Resistance training in cardiac rehabilitation. J. Cardiopulm Rehabil..

[4] Burd N.A., West D.W., Staples A.W., Atherton P.J., Baker J.M., Moore D.R., Holwerda A.M., Parise G., Rennie M.J., Baker S.K. (2010). Low-load high volume resistance exercise stimulates muscle protein synthesis more than high-load low volume resistance exercise in young men. PLoS ONE..

[5] Hurley B.F., Roth S.M. (2000). Strength training in the elderly: Effects on risk factors for age-related diseases. Sports Med..

[6] Delagardelle C., Feiereisen P. (2005). Strength training for patients with chronic heart failure. Eura Medicophys..

[7] Pepera G., Christina M., Katerina K., Argirios P., Varsamo A. (2021). Effects of multicomponent exercise training intervention on hemodynamic and physical function in older residents of long-term care facilities: A multicenter randomized clinical controlled trial. J. Bodyw. Mov. Ther..

[8] Garza M.A., Wason E.A., Cruger J.R., Chung E., Zhang J.Q. (2019). Strength training attenuates post-infarct cardiac dysfunction and remodeling. J. Physiol. Sci..

[9] Overend T., Versteegh T., Thompson E., Birmingham T., Vandervoort A. (2000). Cardiovascular stress associated with concentric and eccentric isokinetic exercise in young and older adults. J. Gerontol. Seri. A..

[10] Stöhr E., Stembridge M., Shave R., Samuel J., Stone K., Esformes J. (2017). Systolic and diastolic LV mechanics during and following resistance exercise. Med. Sci. Sports Exerc..

[11] Howlett L.A., O’Sullivan K., Sculthorpe N., Richards J. (2020). The effect of varying intensities of lower limb eccentric muscle contractions on left ventricular function. Eur. J. Appl. Physiol..

[12] Cheetham C., Green D., Collis J., Dembo L., O’Driscoll G. (2002). Effect of aerobic and resistance exercise on central hemodynamic responses in severe chronic heart failure. J. Appl. Phisiol..

[13] Gjøvaag T.F., MIrtaheri P., Simon K., Berdal G., Tuchel I., Westle T., Bruusgaard K.A., Nilsson B.B., Hisdal J. (2016). Hemodynamic Responses to Resistance Exercise in Patients with Coronary Artery Disease. Med. Sci. Sports Exerc..

[14] Kambic T., Hadžić V., Lainscak M. (2021). Hemodynamic Response to High- and Low-Load Resistance Exercise in Patients with Coronary Artery Disease: A Randomized, Crossover Clinical Trial. Int. J. Environ. Res. Public Health.

[15] Lamotte M., Chevalier A., Jamon A., Brassine E., Van de Borne P. (2009). Hemodynamic response of an isokinetic testing and training session. Isokinet. Exerc. Sci..

[16] Wright S., Esfandiari S., Elmayergi N., Sasson Z., Goodman J.M. (2014). Left atrial functional changes following short-term exercise training. Eur. J. Appl. Physiol..

[17] Henein M.Y., Cameli M., Lindqvist P., Wiklund U., Mandoli G.E., Mondillo S. (2018). Peak Atrial Longitudinal Strain (PALS): Better Call it Stretch?. Int. Cardiovasc. Forum J..

[18] Santoro A., Alvino F., Antonelli G., Molle R., Mondillo S. (2016). Left atrial strain after maximal exercise in competitive waterpolo players. Intern. J. Cardiovasc. Imaging.

[19] Sareban M., Winkert K., Berger M.M., Niebauer J., Steinacker J.M., Treff G. (2018). Speckle tracking-derived bi-atrial strain before and after eleven weeks of training in elite rowers. Sci. Rep..

[20] Sugimoto T., Bandera F., Alfonzetti E., Bussadori C., Guazzi M. (2017). Left atrial function dynamics during exercise in heart failure pathophysiological implications on the rightheart and exercise ventilation inefficiency. JACC Cardiovasc. Imaging.

[21] Brown L.E., Weir J.P. (2001). ASEP Procedures Recommendation I: Accurate Assessment of Muscular Strength and Power. J. Exerc. Physiol..

[22] Brzycki M. (1993). Strength Testing—Predicting a One-Rep Max from Reps-to-Fatigue. J. Phys. Educ. Recreat. Dance.

[23] Stöhr E.J., Stembridge M., Esformes J.I. (2015). In vivo human cardiac shortening and lengthening velocity is region dependent and not coupled with heart rate: ‘longitudinal’ strain rate markedly underestimates apical contribution. Exp. Physiol..

[24] Borg G.A. (1982). Psychophysical bases of perceived exertion. Med. Sci. Sports Exerc..

[25] Kasner M., Sinning D., Burkhoff D., Tschöpe C. (2015). Diastolic pressure-volume quotient (DPVQ) as a novel echocardiographic index for estimation of LV stiffness in HFpEF. Clin. Res. Cardiol..

[26] Machino-Ohtsuka T., Seo Y., Tada H., Ishizu T., Machino T., Yamasaki H., Igarashi M., Xu D., Sekiguchi Y., Aonuma K. (2011). Left atrial stiffness relates to left ventricular diastolic dysfunction and recurrence after pulmonary vein isolation for atrial fibrillation. J. Cardiovasc. Electrophysiol..

[27] Fukuta H., Little W.C. (2008). The cardiac cycle and the physiologic basis of left ventricular contraction, ejection, relaxation, and filling. Heart Fail. Clin..

[28] Nishikawa Y., Roberts J.P., Tan P., Klopfenstein C.E., Klopfenstein H.S. (1994). Effect of dynamic exercise on left atrial function in conscious dogs. J. Physiol..

[29] Cuspidi C., Tadic M., Sala C., Gherbesi E., Grassi G., Mancia G. (2019). Left atrialfunction in elite athletes:A meta-analysis of two-dimensional speckle tracking echocardiographic studies. Clin. Cardiol..

[30] Tan Y.T., Wenzelburger F., Lee E., Nightingale P., Heatlie G., Leyva F., Sanderson J.E. (2010). Reduced left atrial function on exercise in patients with heart failure and normal ejection fraction. Heart.

[31] Lentini A.C., Mckelvie R.S., McCartney N., Tomlinson C.W., MacDougall J.D. (1993). Left ventricular response in healthy young men during heavy-intensity weight-lifting esercise. J. Appl. Physiolog..

[32] Park J.H., Marwick T.H. (2011). Use and Limitations of E/e’ to Assess Left Ventricular Filling Pressure by Echocardiography. J. Cardiovasc. Ultrasound..

[33] Andersen O.S., Smiseth O.A., Dokainish H., Abudiab M.M., Schutt R.C., Kumar A., Sato K., Harb S., Gude E., Remme E.W. (2017). Estimating Left Ventricular Filling Pressure by Echocardiography. J. Am. Coll. Cardiol..

[34] Pathan F., D’Elia N., Nolan M.T., Marwick T.H., Negishi K. (2017). Normal Ranges of Left Atrial Strain by Speckle-Tracking Echocardiography: A Systematic Review and Meta-Analysis. J. Am. Soc. Echocardiogr..

[35] Anwar A.M., Geleijnse M.L., Soliman O.I., Nemes A., ten Cate F.J. (2007). Left atrial Frank-Starling law assessed by real-time, three-dimensional echocardiographic left atrial volume changes. Heart.

[36] Caminiti G., Iellamo F., Perrone M.A., D’Antoni V., Catena M., Manzi V., Morsella V., Franchini A., Volterrani M. (2021). Central Hemodynamic Adjustments during Post-Exercise Hypotension in Hypertensive Patients with Ischemic Heart Disease: Concurrent Circuit Exercise versus High-Intensity Interval Exercise. A Preliminary Study. J. Clin. Med..

[37] Zile M.R., Kjellstrom B., Bennett T., Cho Y., Baicu C.F., Aaron M.F., Abraham W.T., Bourge R.C., Kueffer F.J. (2013). Effects of exercise on left ventricular systolic and diastolic properties in patients with heart failure and a preserved ejection fraction versus heart failure and a reduced ejection fraction. Circ. Heart Fail..

[38] Andersen M.J., Ersbøll M., Bro-Jeppesen J., Gustafsson F., Hassager C., Køber L., Borlaug B.A., Boesgaard S., Kjærgaard J., Møller J.E. (2012). Exercise hemodynamics in patients with and without diastolic dysfunction and preserved ejection fraction after myocardial infarction. Circ. Heart Fail..

[39] Stefani L., Toncelli L., Di Tante V., Vono M., Cappelli B., Pedrizzetti G., Galanti G. (2008). Supernormal functional reserve of apical segments in elite soccer players: An ultrasound speckle tracking handgrip stress study. Cardiovas. Ultrasound.

[40] Weiner R.B., Weyman A.E., Kim J.H., Wang T.J., Picard M.H., Baggish A.L. (2012). The impact of isometric handgrip testing on left ventricular twist mechanics. J. Physiol..

[41] MacDougall J.D., Tuxen D., Sale D.G., Moroz J.R., Sutton J.R. (1985). Arterial blood pressure response to heavy resistance exercise. J. Appl. Physiol..

